# Crossing the Blood–Brain Barrier: Innovations in Receptor- and Transporter-Mediated Transcytosis Strategies

**DOI:** 10.3390/pharmaceutics17060706

**Published:** 2025-05-28

**Authors:** Ling Ding, Pratiksha Kshirsagar, Prachi Agrawal, Daryl J. Murry

**Affiliations:** 1 Clinical Pharmacology Laboratory, Department of Pharmacy Practice and Science, University of Nebraska Medical Center, Omaha, NE 68198, USA; ling.ding@unmc.edu (L.D.); pkshirsagar@unmc.edu (P.K.); pagrawal@unmc.edu (P.A.); 2 Fred and Pamela Buffett Cancer Center, University of Nebraska Medical Center, Omaha, NE 68198, USA

**Keywords:** brain delivery, transcytosis, blood–brain barrier, nanomaterials

## Abstract

The blood–brain barrier (BBB) is a highly selective and natural protective membrane that restricts the entry of therapeutic agents into the central nervous system (CNS). This restrictive nature poses a major challenge for pharmacological treatment of a wide range of CNS disorders, including neurodegenerative disorders, brain tumors, and psychiatric conditions. Many chemical drugs and biopharmaceuticals are unable to cross the BBB, and conventional drug delivery methods often fail to achieve sufficient brain concentrations, leading to reduced therapeutic efficacy and increased risk of systemic toxicity. In recent years, targeted drug delivery strategies have emerged as promising approaches to overcome the BBB and enhance the delivery of therapeutic agents to the brain. Among these, receptor-mediated transcytosis (RMT) and transporter-mediated transcytosis (TMT) are two of the most extensively studied mechanisms for transporting drugs across brain endothelial cells into the brain parenchyma. Advances in materials science and nanotechnology have facilitated the development of multifunctional carriers with optimized properties, improving drug targeting, stability, and release profiles within the brain. This review summarizes the physiological structure of the BBB and highlights recent innovations in RMT- and TMT-mediated brain drug delivery systems, emphasizing their potential not only to overcome current challenges in CNS drug development, but also to pave the way for next-generation therapies that enable more precise, effective, and personalized treatment of brain-related diseases.

## 1. Introduction

Brain disease, including brain cancers and central nervous system (CNS) disorders, are among the most prevalent, devastating and inadequately treated conditions. Developing safe and effective therapeutic strategies for CNS malignancies presents significant challenges, including high failure rates and extended timelines to reach the market compared to non-CNS therapies. Notably, the U.S Food and Drug Administration (FDA) has approved several drugs for utilization for the treatment of brain tumors, such as everolimus, bevacizumab, carmustine, dabrafenib mesylate, temozolomide, trametinib dimethyl sulfoxide, and vorasidenib citrate [1]. A critical obstacle in the development of CNS therapies is the blood–brain barrier (BBB), initially identified by Paul Ehrlich in the late 19th and early 20th centuries. Ehrlich’s observation that dyes injected into the bloodstream failed to stain brain tissue underscored the barrier’s selective permeability [2].

The BBB is a highly specialized and semipermeable structure that separates the CNS from the systemic circulation. It plays a crucial role in maintaining the brain microenvironment. The BBB acts as both a protective shield and a regulator, ensuring the CNS is isolated from toxins, pathogens, and potentially harmful fluctuations in blood composition, such as hormones or metabolites that could disrupt normal function [3]. By maintaining a tightly controlled environment, the BBB is essential for preserving neuronal activity and overall brain homeostasis.

Structurally, the BBB is composed of tightly bound endothelial cells (ECs) that line the brain’s capillaries. These ECs are reinforced by surrounding pericytes, astrocytes, and other supporting elements in the extracellular matrix (ECM) [4]. Pericytes and astrocytes not only provide structural support, but also contribute to the regulation of BBB permeability and respond to physiological or pathological changes in the brain. They are crucial for the development of accurate in vitro models of the BBB [5]. Other cellular and molecular components, such as enzymes and efflux transporters like P-glycoprotein (P-gp) and breast cancer resistance protein (BCRP), further enhance the barrier’s protective function by actively removing harmful substances [6]. Efflux transporters are proteins embedded in cell membranes that actively transport molecules out of the cells, essentially acting like “pumps” to remove unwanted substances. The BBB’s complex architecture and stringent selective permeability significantly hinder drug delivery to the CNS, excluding over 95% of drug candidates. The BBB allows the passive diffusion of only lipophilic molecules with a molecular weight under 400 Da and specific drugs with favorable physicochemical properties. Additionally, active transport systems facilitate the controlled entry of essential nutrients, such as glucose, amino acids, and ions required for neural metabolism [7,8]. However, large molecules, hydrophilic compounds, and many therapeutic agents, such as antibody–drug conjugate (ADC), are excluded, limiting the ability of systemic treatment to reach the brain. This exclusion becomes even more challenging in pathological conditions like CNS tumors. The progression of disease leads to the formation of a blood–tumor barrier (BTB), which imposes additional constraints on achieving effective drug concentrations within the tumor microenvironment [9].

In recent years, drug development for brain diseases has advanced substantially, with a particular focus on the utilization of polymeric and lipid-based nanoparticles (NPs) to enhance drug delivery to the brain. Nanocarriers can be modified to optimize brain targeting, enhance stability, and modify drug-release patterns. Despite these efforts, the success rate of CNS drug development remains among the lowest in the pharmaceutical industry, primarily due to the challenges posed by the BBB and BTB [7,8]. Effective and efficient drug delivery systems capable of traversing this protective barrier are indispensable for the development of therapies targeting CNS disorders. These systems must not only transport therapeutic agents across the BBB, but also ensure targeted delivery to specific brain regions, minimize systemic toxicity, and optimize pharmacokinetics and pharmacodynamics [10]. Lipid-based NPs, such as liposomes, lipid nanoparticles (LNPs), nanostructured lipid carriers (NLCs), solid lipid nanoparticles (SLNs), and emulsions, have emerged as promising carriers for drug delivery [11]. Their structural properties, including a lipid bilayer or lipid matrix, mimic biological membranes, facilitating the crossing of the BBB. Lipid-based NPs can encapsulate a wide range of drugs, including small molecules, peptides, and nucleic acids, protecting them from degradation and enabling sustained release, making them more attractive [12]. However, the potential immune reactions to NPs must be carefully considered and managed [13]. Polymeric NPs, including micelles, polyplexes, and polymeric hydrogels, offer additional advantages, such as a high drug-loading capacity, tunable release profiles, and structural versatility [14]. These NPs have been successfully used in preclinical and clinical research, delivering drugs for CNS diseases including medulloblastoma (MB), Alzheimer’s disease (AD), Parkinson’s disease, multiple sclerosis, and stroke [15]. One of the most notable advantages of NP-based systems is their ability to encapsulate drugs into nanocarriers, thereby facilitating sustained drug release. This capability not only reduces the dosing frequency, but also enhances patient compliance and optimizes therapeutic outcomes by ensuring that drug concentrations remain within the therapeutic window [16,17].

Surface modifications of NPs further enhance their functionality and ability to cross the BBB. Functionalization with targeting ligands, such as transferrin, lactoferrin, insulin, or low-density lipoprotein (LDL) receptors, facilitates receptor-mediated transcytosis (RMT) across the BBB [18]. Additionally, modifying NPs with cell-penetrating peptides or specific antibodies can improve their interaction with BBB ECs and promote selective delivery to the brain [19]. Transporter-mediated transcytosis (TMT), another promising strategy, exploits endogenous transport systems, such as glucose or amino acid transporters, to deliver drugs effectively into the brain [20]. TMT routes across the BBB include multiple mechanisms, each with unique challenges and opportunities for therapeutic delivery. For the BBB, the paracellular route allows water-soluble drugs to pass through tight junctions between ECs, though this pathway is highly restricted due to the barrier’s tightness [4]. The transcellular lipophilic pathway enables small, lipophilic molecules to diffuse across ECs, but limits the range of molecules that can be delivered [21]. Advanced strategies focus on exploiting active transport mechanisms such as RMT and TMT. RMT uses ligand–receptor interactions to transport macromolecules such as NPs, proteins, or peptides across the BBB, while TMT leverages transport proteins to ferry small molecules and nutrients [22]. Furthermore, adsorption-mediated transcytosis (AMT), driven by electrostatic interactions between positively charged NPs and negatively charged cell membranes, offers an alternative route for delivering therapeutics to the brain. However, AMT remains less specific than RMT or TMT, which limits its utility [23].

Ongoing research into the anatomical and pathological characteristics of the BBB is crucial for enhancing these strategies. Advancements in understanding how BBB integrity alters under pathological conditions provide insights into developing targeted therapies. Current research focuses on developing NPs with ligands or other modifications that can specifically bind to receptors on the BBB or/and the targeted cells to facilitate CNS drug delivery. While promising in preclinical studies, the translation of NP-based CNS drug delivery to clinical trials can be challenging due to safety concerns and complex formulation considerations. In this review, the structure and physiology of the BBB will be summarized, accompanied by an in-depth discussion of various RMT and TMT mechanisms for crossing the BBB. Particular attention will be given to the emerging roles of RMT and TMT as innovative strategies for overcoming the BBB, as well as recent advancements in drug delivery over the past five years in the treatment of CNS disorders. Through such focused research, novel approaches for crossing the BBB and achieving effective brain accumulation of therapeutics can be realized, ultimately addressing the unmet medical needs of CNS diseases.

## 2. BBB Structure and Physiology

**Figure 1:**
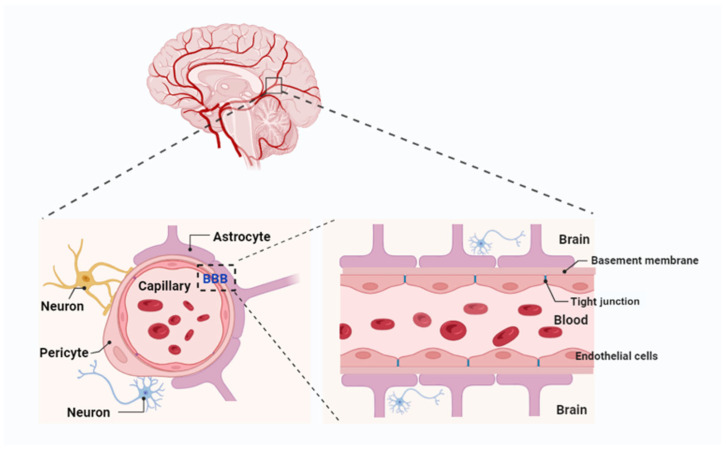
Blood–brain barrier (BBB) components and composition. The BBB is composed of endothelial cells with tight junctions, supported by a basement membrane, astrocyte endfeet, pericytes, and extracellular matrix components, with its major functional site located in the capillaries. Created with Biorender.com.

The BBB is a complex and dynamic structure, primarily composed of ECs with tight junctions, supported by a basement membrane, astrocytes, pericytes, and extracellular matrix (ECM) components. Its major functional site is located in the capillaries (Figure 1) [4]. The BBB is considered the largest interface for blood–brain exchange, as it has a combined surface area of 12 and 18 m^2^ per average adult, based on an average microvessel surface area of 150 and 200 cm^2^ per gram of tissue [24]. Additionally, the blood–cerebrospinal fluid barrier (BCSFB) [25] and the arachnoid barrier represent the second and third barriers in the brain. However, the arachnoid barrier’s contribution to the exchange between the blood and the brain is insignificant, due to its avascular nature and relatively small surface area compared to the BBB and BCSFB [26].

The function of the BBB is contingent upon the coordinated contributions of multiple cell types within the neurovascular unit (NVU). This unit comprises astrocytes, pericytes, neurons, and microglia, all of which work together to maintain the integrity and selective permeability of the BBB [27]. Astrocytes, with their extensive “endfeet” covering 99% of the basal capillary membrane, play a vital role in the development and maintenance of the BBB by secreting factors such as GDNF, angiopoietin-1, and angiotensin II. These factors support EC integrity and regulate barrier function. Pericytes, which encircle ECs, provide structural support, regulate blood flow, and limit barrier permeability. Recent research highlights the location-dependent diversity of pericytes: those adjacent to arterioles regulate blood flow through contractile properties, while venous capillary pericytes maintain barrier integrity and modulate immune responses. These interactions between pericytes, astrocytes, and ECs are critical for preserving BBB integrity. Studies have demonstrated that pericyte deficiency increases BBB permeability, contributing to neurovascular dysfunction in diseases such as Alzheimer’s, stroke, and multiple sclerosis [28]. Consequently, these cell types form a highly coordinated system that safeguards the brain while facilitating the regulated exchange of nutrients and waste products.

The ECs that form the BBB are tightly joined by specialized junctions known as tight junctions (TJs), composed of transmembrane proteins such as claudins, occludins, and junctional adhesion molecules (JAMs) [29]. These proteins create a high-resistance paracellular barrier that limits the crossing of ions and molecules while ensuring selective permeability to essential substances such as oxygen and glucose. Claudins, particularly claudin-5, are crucial for maintaining BBB integrity; their disruption has been linked to various neurological disorders [30]. Additionally, ECs exhibit limited pinocytotic activity and express efflux transporters [31]. Efflux transporters in brain capillary ECs further enhance the BBB’s protective function by actively removing undesirable substances and metabolic byproducts from the brain into the systemic circulation. Key players include multidrug resistance transporters like P-glycoprotein, multidrug resistance proteins (MRPs), and BCRP [32]. While these systems safeguard the CNS, they also hinder the delivery of many therapeutic agents. Drugs designed to treat neurological disorders are often recognized as substrates and removed from the brain before achieving therapeutic levels. To address this, researchers are exploring strategies such as efflux transporter inhibitors, drug designs that evade transporter recognition, and advanced delivery systems. These approaches aim to balance the dual role of efflux transporters—protecting the brain while enabling effective drug delivery—without compromising the integrity or function of the BBB.

## 3. Transcytosis

Transcytosis is a fundamental transcellular transport mechanism that enables the internalization of molecules by endosomes on one side of a cell. These endosomes subsequently transport the molecules across the cytoplasm for release on the other side. Unlike paracellular transport, which occurs between cells, transcytosis facilitates the direct movement of molecules through a cell [33]. This process comprises three key steps: (1) internalization, where molecular uptake occurs via endocytosis, forming endosomes that encapsulate the cargo; (2) transport, where the endosomes traverse the cytoplasm, shuttling molecules across the cell membrane; and (3) release, where the endosomes fuse with the cell membrane on the opposite side, expelling their contents via exocytosis. At the BBB, transcytosis plays a pivotal role in transport across brain ECs. Given the BBB’s restrictive nature, this process is essential for delivering substances that cannot passively diffuse into the brain, ensuring the maintenance of brain homeostasis and neuronal function. Understanding transcytosis is vital for developing drug delivery strategies and therapeutics aimed at overcoming the BBB’s selective permeability. The regulation of endosomal trafficking plays a pivotal role in optimizing TMT and RMT efficiency at the BBB. This regulation influences the overall effectiveness of drug delivery strategies that target transcytosis pathways.

## 4. Mechanisms of BBB Crossing by Nanomaterials

Nanomaterials employ various mechanisms to cross the BBB, facilitating the targeted delivery of therapeutics to the brain. These mechanisms can be broadly categorized into passive and active transport pathways. Passive diffusion occurs when small, lipophilic nanomaterials cross the BBB without the involvement of specific transporters. In contrast, active transport mechanisms encompass TMT or carrier-mediated transport (CMT), RMT, and AMT. TMT exploits endogenous transporters expressed on brain ECs to actively shuttle molecules across the BBB, leveraging physiological pathways responsible for the transport of essential nutrients, peptides, and ions between the bloodstream and brain [3]. RMT relies on ligands, peptides, or antibodies conjugated to nanomaterials that engage specific receptors, such as transferrin receptors (TfRs) or low-density lipoprotein receptor-related proteins (LRP1 and LRP2), to facilitate endocytosis and transcytosis across the BBB [34]. AMT is mediated by electrostatic interactions between positively charged nanomaterials with negatively charged membrane components, promoting cellular uptake [12]. Beyond these traditional approaches, emerging strategies, such as cell-mediated transport via monocytes or exosomes, and stimuli-responsive nanomaterials that react to pH, temperature, or enzymatic activity, are being explored to enhance BBB permeability and improve therapeutic efficacy. These mechanisms can be further classified into invasive or non-invasive approaches. Invasive approaches involve the temporary disruption of the BBB through physical means, enabling nanomaterials to cross the BBB via the paracellular pathway. Techniques such as focused ultrasound-mediated BBB opening and local delivery strategies fall under this category, which is also referred to as the paracellular pathway [35,36]. Conversely, non-invasive strategies maintain BBB integrity during drug delivery and rely on the transcellular pathway to improve nanomaterial transport. TMT, RMT, and AMT, as well as emerging cell-mediated and stimuli-responsive nanomaterials, fall within this category, and can be collectively to referred to as transcellular mechanisms. These diverse transport mechanisms offer promising strategies for overcoming the restrictive nature of the BBB, thereby offering novel opportunities for the treatment of neurological disorders.

## 5. Transporter-Mediated Transcytosis (TMT) for Crossing the BBB

**Figure 2:**
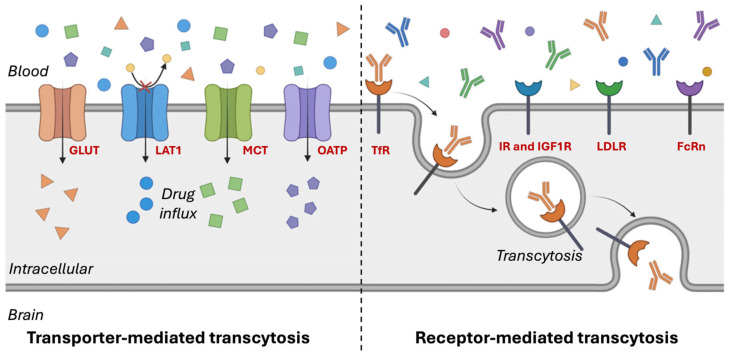
Transporter- and receptor-mediated transcytosis in endothelial cells. Created with Biorender.com. Glucose transporters (GLUTs); L-type amino acid transporter 1 (LAT1); monocarboxylate transporters (MCTs); organic anion-transporting polypeptides (OATPs); transferrin receptors (TfRs); insulin receptors (IRs); insulin-like growth factor-1 receptor (IGF1R); low-density lipoprotein receptors (LDLRs); neonatal Fc receptor (FcRn).

Among the various strategies to overcome the BBB, TMT has gained significant attention due to its potential to facilitate efficient drug delivery to the CNS. TMT exploits specific endogenous transporters expressed on the ECs of the BBB to actively transport molecules across this tightly regulated barrier. By leveraging the natural mechanisms that transport essential nutrients, peptides, and ions between the bloodstream and brain, TMT enables efficient and targeted drug delivery to the brain (Figure 2) [37,38]. These TMT transporters are encoded by genes within the solute carrier (SLC) transporter gene family, which comprises over 300 genes that regulate the translocation of various substrates across the BBB [39]. Key transporters involved in TMT include glucose transporters (GLUTs), which mediate glucose and mannose uptake; neutral amino acid transporters (LAT1 and LAT2), responsible for transporting large neutral amino acids and certain drugs like L-dopa; and anionic/cationic amino acid transporters, which facilitate the transport of aspartate, glutamate, L-lysine, L-arginine, and L-ornithine [38]. Additionally, monocarboxylate transporters (MCTs) play a critical role in lactate transport, while lipid transporters, such as LRP1, facilitate the translocation of lipoproteins and other macromolecules. Dipeptide transporters such as PEPT2 also contribute to TMT by shuttling di- and tripeptides across the BBB [40,41,42].

The polarized distribution of transporters across the BBB contributes to its functional asymmetry. Certain transport proteins are exclusively localized to either the luminal or abluminal membrane, while others are expressed on both membranes of the ECs [43]. By harnessing these transport pathways, targeted drug delivery strategies can be developed to facilitate the efficient transport of therapeutic agents that would otherwise be excluded from the brain. Recent advancements in TMT-based drug delivery have focused on two primary approaches: (1) designing drug molecules that structurally mimic endogenous substrates of specific transporters, and (2) conjugating therapeutic compounds or nanocarriers with ligands that selectively bind to and engage transporter-mediated pathways [44]. TMT-based drug delivery systems have demonstrated significant potential in enhancing brain uptake of poorly permeable therapeutics, particularly through strategies such as LAT1-targeted prodrugs and GLUT1-based delivery platforms [45,46]. These innovative strategies improve the selective passage of therapeutics across the restrictive BBB, while preserving its structural integrity.

### 5.1. Glucose Transporters (GLUTs)

GLUTs, encoded by SLC2A1, play a crucial role in facilitating glucose transport across the BBB to meet the brain’s high energy demands [47,48]. Among the 14 identified human GLUT isoforms, GLUT1 to GLUT5 are the most extensively characterized, primarily transporting hexoses, notably D-glucose, while also facilitating the uptake of other hexoses, such as D-mannose and D-galactose, albeit with lower efficiency [48,49,50,51]. As a facilitative transporter, GLUT1 mediates passive glucose diffusion and is highly expressed on both the luminal (blood-facing) and abluminal (brain-facing) membranes of BBB ECs, ensuring a continuous glucose supply to the brain [52]. Unlike sodium-dependent glucose transporters (SGLTs), which require ion co-transport, GLUT1 functions independently of sodium gradients, allowing for energy-independent glucose uptake [53]. Beyond its fundamental physiological role, GLUT1 expression is tightly regulated in response to metabolic demands, and can be altered under pathological conditions [54]. In neurodegenerative disorders such as AD, decreased GLUT1 expression impairs glucose transport to the brain, contributing to cognitive decline [55]. Conversely, in glioblastoma and other malignancies, GLUT1 and GLUT3 cause rapid tumor proliferation by facilitating enhanced glucose uptake [55,56,57,58]. Furthermore, cerebrovascular conditions like ischemia can alter GLUT expression, thereby affecting glucose supply to the brain and further highlighting GLUTs as a potential therapeutic target [58,59].

Ligand-modified nanomaterials that mimic glucose structures or bind to GLUT1-binding domains have been developed to exploit GLUT1-mediated transport, enhancing CNS drug delivery. This understanding has led to an increasing interest in targeting GLUT1 for disease intervention and drug delivery. Recent studies have demonstrated that GLUT1-targeted NPs can improve the delivery of chemotherapeutic agents, neuroprotective peptides, and genetic material to the brain, leading to improved therapeutic efficacy [60]. By harnessing GLUT1-mediated transport, researchers seek to enhance drug bioavailability and optimize treatment strategies for brain-related diseases [61]. Given its critical role in BBB function and disease pathophysiology, GLUT1 remains a promising target for CNS drug delivery and therapeutic intervention [62].

One approach involved modifying liposomal NPs with mannose, a known GLUT1 ligand, to facilitate targeted delivery in AD treatment [60]. This strategy significantly enhanced brain-derived neurotrophic factor (BDNF) transport, achieving approximately 50% higher BBB permeability and a ~1.7-fold increase in BDNF levels in neuronal cells [60]. Similarly, Xie et al. developed glucose-modified liposomes incorporating phospholipids, glucose-derived cholesterols, and polyethylene glycol (PEG) linkers to optimize brain-targeting efficiency [63]. In one study, liposomes incorporating various glucose-conjugated cholesterols (GLU200-LIP, GLU1000-LIP, and GLU2000-LIP) demonstrated promising brain-targeting capabilities [63]. In addition, novel multivalent glucoside-based ligands with strong affinity for GLUT1 have been developed for brain-targeted liposomal drug delivery. Liposomes functionalized with these glucosides and encapsulated with docetaxel achieved significantly higher brain accumulation of docetaxel compared to both free docetaxel and non-targeted liposome formulations [64]. Xie et al. designed dual-sensitive nanomicelles functionalized with glucose molecules to enhance brain delivery of 3D6 antibody fragments (3D6-Fab) via GLUT1-mediated transport [65]. This strategy achieved a 41-fold increase in brain accumulation of 3D6-Fab, representing a promising platform for efficient delivery of therapeutic antibodies to the CNS for the treatment of neurological diseases. Further advancing GLUT1-mediated transcytosis, Hao et al. introduced sequential targeting in crosslinking (STICK) NPs, a polymer-based delivery system designed to enhance both BBB penetration and tumor targeting [66]. By incorporating maltobionic acid (a GLUT1-recognized glucose derivative) and 4-carboxyphenylboronic acid, this system improved drug stability, facilitated BBB transport, and significantly inhibited tumor growth in preclinical models of aggressive brain tumors [66,67]. Additionally, Zhou et al. developed glycosylated “triple-interaction” stabilized polymeric siRNA NPs (Gal-NP@siRNA) to target β-site amyloid precursor protein cleaving enzyme 1 (BACE1) in an amyloid precursor protein (APP)/PS1 transgenic AD mouse model [68]. These NPs demonstrated superior blood stability and efficiently penetrated the BBB via glycemia-controlled GLUT1-mediated transport. Once in the brain, the siRNAs effectively reduced BACE1 expression, modulating key pathological pathways in AD progression (Table 1) [68].

These findings highlight the significant potential of GLUT1-targeted nanocarriers in CNS drug delivery. They demonstrate enhanced therapeutic efficacy through improved BBB transport, increased drug stability, and targeted disease modulation. However, challenges persist, particularly in studying human GLUTs in vivo, necessitating reliable in vitro models [61]. Future research is needed to optimize ligand specificity, enhance transcytosis efficiency, and minimize off-target effects. Additionally, inter-individual variability in GLUT1 expression poses a challenge for clinical translation. Addressing these issues will be key to advancing GLUT1-mediated drug delivery for neurological disorders, brain tumors, and cerebrovascular diseases, paving the way for more effective CNS therapeutics.

### 5.2. L-Type Amino Acid Transporters (LATs)

L-type amino acid transporter 1 (LAT1) is a high-affinity, sodium-independent transporter that facilitates the uptake of large neutral amino acids, including leucine, phenylalanine, tryptophan, and tyrosine [74]. Forming a heterodimeric complex with 4F2 heavy chain (SLC3A2), LAT1 is highly expressed in metabolically active tissues, such as the brain, placenta, testes, and various cancers, supporting protein synthesis, cell proliferation, and metabolic function [74]. At the BBB, LAT1 is primarily located on the luminal membrane of ECs, ensuring the continuous influx of essential amino acids for neuronal and glial function [75]. LAT1 is overexpressed in several cancers, including glioblastoma [76], brain metastases [77], and various solid tumors, as well as in neurological disorders, such as autism spectrum disorders [78]. Additionally, LAT1 facilitates the transport of CNS-active drugs, such as melphalan [79], levodopa (L-Dopa) [80], gabapentin [81], and pregabalin [82], making it integral for developing prodrugs with enhanced BBB permeability [83]. For example, LAT1 has been shown to exhibit a high maximal transport capacity (Vmax ≈ 40–60 nmol/min/g) and a substantial binding affinity (Km ≈ 10–200 µM), enabling the rapid exchange of high-affinity substrates across the BBB with half-times (half the time required to reach drug concentration equilibrium between the brain and blood) under 15 min [83].

Recent advancements in LAT1-targeted drug delivery have focused on designing prodrugs and NPs that exploit LAT1-mediated transport. Montaser et al. developed LAT1-targeted prodrugs to enhance the delivery of nonsteroidal anti-inflammatory drugs (NSAIDs) to the brain. Salicylic acid derivatives exhibited a five-fold increase in brain uptake, but challenges such as plasma protein binding and premature bioconversion remain [84]. Similarly, ferulic acid (FA)-based prodrugs have demonstrated LAT1-specific binding, enabling BBB penetration and cellular uptake. These amide-based prodrugs, incorporating an aromatic ring in the moiety, exhibited effective binding to LAT1, facilitating their cellular uptake in vitro and successfully crossing the BBB in mice [85]. Beyond small molecules, LAT1-targeted NPs have shown promise. L-DOPA-functionalized gold nanoflowers (L-DOPA-AuNFs) exhibited superior BBB permeability and uptake by brain macrophages without inducing inflammation [86]. In the context of glioma therapy, Zhang et al. developed LAT1-targeted liposomes co-loaded with temozolomide (TMZ) and sorafenib [87]. The high expression of LAT1 on BBB and glioma cells enabled these liposomes to cross the BBB more efficiently, enhancing drug delivery to the tumor site and improving therapeutic outcomes (Table 2) [87].

Given its broad substrate specificity, high transport efficacy, and key role in both CNS and tumor metabolism, LAT1 represents a powerful avenue for brain-targeted drug delivery [88]. Ongoing research into LAT1-mediated strategies, including optimized ligand conjugation, prodrug refinement, and NP engineering, holds promise for enhancing therapeutic outcomes in CNS diseases and brain tumors. By leveraging its strategic expression in key disease areas, LAT1 presents a critical opportunity for advancing more precise and effective treatments for complex neurological and oncological conditions. The following section delves into recent advancements based on LAT1-targeted strategies for overcoming the BBB.

**Table 2:** Strategies to target L-type amino acid transporter 1 (LAT1) for brain-targeted drug delivery.

Drug	Malignancy	LAT1 Targeting Moiety	Delivery Vehicle	Inference	Reference
Salicylic acid, ibuprofen, naproxen, and flurbiprofen	Neurodegenerative diseases	L-Phenylalanine	Prodrug	Significant (5-fold) increase in brain uptake across BBB	[84]
Ferulic acid	Alzheimer’s disease	L-Phenylalanine	Prodrug	Amide-based prodrug with aromatic ring effectively binds to LAT1, facilitating cellular uptake in vitro and crossing BBB	[84]
Levodopa	Brain malignancies like Parkinson’s disease	L-Phenylalanine	L-DOPA-functionalized gold NPs	Significantly higher penetration across BBB, increased internalization in brain macrophages, and inflammatory responses as compared to non-targeted NPs	[86]
Temozolomide and sorafenib	Glioblastoma	Tyrosine	Lipid NPs (carboxylated polyethylene glycol stearate and PLGA-PEG)	Enhanced drug delivery across BBB, increased tumor accumulation, and improved anti-tumor efficacy	[87]
Morin hydrate	Alzheimer’s disease	Phenylalanine-phenylalanine dipeptide	Dipeptide-functionalized PLGA NPs	Significantly improved brain retention with liver and lung accumulation	[89]
Antisense oligonucleotide	Neurodegenerative diseases	Phenylalanine	Phenylalanine-functionalized PEG-PLL NPs	Sixty-four-fold higher brain accumulation when compared to non-targeted NPs	[90]
Saxagliptin	Alzheimer’s disease	L-valine-conjugated chitosan	Chitosan-L-valine NPs	Enhanced BBB permeability with ~50-fold higher brain uptake than free drug and 3.4-fold lower plasma concentration, suggesting stability in plasma and release in brain tissue	[91]
Levodopa and dopamine	Parkinson’s disease		Polyvinylpyrrolidone-functionalized selenium NPs	Facilitated BBB permeability and efficient internalization in brain endothelial cells	[92]
Phenylalanine analogs	Glioblastoma multiforme	Phenylalanine	Free drug	Iodine substitution at second position improved LAT1 affinity, but reduced velocity; reducing one carbon reduced LAT1 affinity, and bicyclic and *α*-methyl phenylalanine showed similar velocity, with preferential tumor accumulation of bicyclic phenylalanine	[93]

Poly(lactic-co-glycolic acid) (PLGA); polyethylene glycol (PEG); poly(ethylene glycol)-b-poly(l-lysine) (PEG-PLL).

### 5.3. Monocarboxylate Transporters (MCTs)

MCTs constitute a family of transmembrane proteins responsible for the transport of monocarboxylates, including lactate, pyruvate, ketone bodies (β-hydroxybutyrate and acetoacetate), and short-chain fatty acids, across biological membranes. These proton-coupled symporters facilitate energy-independent transport by co-transporting protons (H^+^) along substrate concentration gradients [40]. Among the MCT family, MCT1 (SLC16A1), MCT2 (SLC16A7), and MCT4 (SLC16A3) hold particular significance in brain metabolism and BBB function.

At the BBB, MCT1 is predominantly expressed on the luminal membrane of endothelial cells, facilitating the uptake of ketone bodies during periods of low glucose availability, such as fasting, exercise, or ketogenic diets [44,94,95]. Additionally, MCT1 plays a crucial role in transporting lactate from the bloodstream into the brain, where it serves as an energy substrate for neurons and glial cells [96]. MCT2, primarily expressed in neurons, supports lactate uptake for metabolic processes, whereas MCT4, found in astrocytes, facilitates lactate export to sustain neuronal energy demands [97]. Given their essential role in brain metabolism, MCTs have emerged as promising targets for drug delivery, particularly in conditions associated with metabolic dysfunction and neurodegeneration.

Taking advantage of the natural substrate affinity of MCT1, researchers have developed MCT1-mediated drug delivery systems, including NPs, prodrugs, and inhibitors, to enhance drug penetration into the brain. For instance, Venishetty et al. designed β-hydroxybutyric acid (HBA)-grafted docetaxel-loaded solid lipid NPs (HD-SLNs) to exploit MCT1-mediated transport, achieving significantly higher brain concentrations of docetaxel than Taxotere^®^ [98]. This system also demonstrated enhanced cellular uptake and controlled drug release, highlighting its potential for CNS drug delivery [98]. Similarly, Güliz Ak et al. developed HBA-conjugated solid lipid NPs (SLNs) for glioblastoma therapy, facilitating the transport of carmustine (BCNU) and TMZ across the BBB [99]. These dual drug-loaded SLNs exhibited controlled release, increased cytotoxicity against glioblastoma cells, and reduced toxicity to healthy cells, making them a promising targeted therapy for glioblastoma multiforme (GBM) [99]. Beyond drug-loaded NPs, inhibitors targeting MCT1 have shown potential in modulating tumor metabolism. Pluronic P85 (P85) NPs were found to inhibit MCT1-mediated lactate transport across brain microvascular ECs without significantly affecting GLUT1 function. This demonstrated a concentration-dependent impact on brain monocarboxylate metabolism, while maintaining safety [100]. In another approach, Huang et al. developed ultra-pH-sensitive NPs encapsulating AZD3965, a selective MCT1 inhibitor [101]. This formulation effectively blocked MCT1 activity in tumors, reversing lactic acid-induced immunosuppression and enhancing the effectiveness of cancer immunotherapy, while reducing systemic toxicity [101]. AZD3965 exhibited rapid oral absorption, high bioavailability, and target engagement with minimal systemic toxicity. However, its potential for brain cancer therapy remains underexplored [102]. The study also revealed nonlinear pharmacokinetics, with dose-dependent increases in exposure and changes in clearance, indicating potential target-mediated drug disposition (TMDD) for AZD3965 [102]. Given its ability to inhibit MCT1 in tumors expressing high MCT1 levels, AZD3965 could enhance drug penetration across the BBB and modulate the tumor microenvironment, warranting further investigation in glioblastoma models (Table 3).

In conclusion, MCT1-targeted drug delivery has demonstrated significant potential for improving brain drug penetration, particularly in glioblastoma and other CNS disorders. The development of HBA-grafted NPs and MCT1 inhibitors such as AZD3965 offers promising avenues for overcoming the BBB limitations and enhancing drug efficacy. However, further research is necessary to optimize these formulations, assess their long-term safety, and expand their clinical applicability. Additionally, more specific studies focusing on MCT1’s role in brain tumors are crucial for refining these strategies and improving therapeutic outcomes for patients with CNS malignancies.

### 5.4. Organic Anion-Transporting Polypeptides (OATPs)

OATPs, a subfamily of the solute carrier (SLC) superfamily, play a critical role in the uptake and distribution of amphipathic molecules, including steroid hormones, bile acids, statins, antihypertensives, antibiotics, antifungals, and chemotherapeutic agents [104]. Their broad tissue distribution and capacity to transport structurally diverse compounds make them key regulators of drug disposition in major organs, including the liver, kidney, intestine, and brain [104,105]. OATP isoforms such as OATP1B1, OATP1B3, OATP1A2, and OATP2B1 are particularly relevant to BBB transport, influencing CNS drug permeability and offering potential targets for enhancing drug delivery to the brain [104,105]. Given their role in modulating drug transport across the BBB, OATPs also represent promising targets for enhancing CNS drug delivery, particularly in neurological disorders, where drug penetration remains a significant challenge.

The expression and function of OATPs are influenced by genetic polymorphisms, age, gender, and dietary factors, contributing to inter-individual variability in drug absorption, distribution, efficacy, and toxicity. Genetic polymorphisms in OATP transporters also impact their functional capacity, leading to variations in pharmacokinetics and pharmacodynamics. Notably, OATP1A4 and OATP1A5 exhibit age- and gender-specific expression patterns, affecting brain drug-transport dynamics [106,107,108]. A deeper understanding of these variations is crucial for optimizing CNS drug delivery strategies, particularly in neurological disorders. Advancements in nanotechnology have enabled the development of OATP-targeted drug delivery systems to enhance CNS drug uptake, offering novel insights into transporter-mediated brain drug delivery, improving BBB penetration and therapeutic efficacy [109,110]. For example, Yang et al. developed OATP2B1-targeted dendrigraft poly-l-lysine-polyethylene glycol NPs for siRNA delivery, demonstrating enhanced targeting precision and gene-silencing efficiency in the mouse brain [109]. Similarly, Reichel et al. designed the HMC-FMX nanoprobe, which facilitated OATP-mediated BBB transport and glioblastoma accumulation, highlighting its potential for brain tumor therapy [110]. Additionally, Thompson et al. found that hypoxia/reoxygenation stress upregulates OATP1A4 expression at the BBB, enhancing atorvastatin transport into the brain, suggesting its potential as a drug delivery target (Table 4) [111].

## 6. Receptor-Mediated Transcytosis (RMT) for Crossing the BBB

RMT is another crucial mechanism that facilitates the transport of macromolecules across the BBB, offering a promising strategy for the delivery of CNS drugs. This process relies on receptor–ligand interactions to selectively transport therapeutics across ECs while maintaining BBB integrity. In this section, we discuss the biological mechanisms of RMT, recent advancements in therapeutic applications, and future directions for optimizing BBB drug delivery. RMT involves three key steps: (1) therapeutic molecules or ligands designed to mimic endogenous substrates bind to specific receptors expressed on the luminal (blood-facing) surface of the ECs; (2) the receptor–ligand complex undergoes vesicular endocytosis, protecting the cargo from degradation and facilitating intracellular transport; and (3) vesicles translocate across ECs and release their cargo into the brain’s interstitial fluid, where the therapeutic agent exerts its effects [115]. Several receptors facilitate RMT across the BBB, including the TfR, insulin receptor (IR), insulin-like growth factor-1 receptor (IGF1R), low-density lipoprotein receptor-related proteins (LRP1 and LRP2), neonatal Fc receptor (FcRn), and angiotensin-converting enzyme receptor (ACE). By targeting receptors involved in the physiological uptake of essential nutrients and signaling molecules, RMT provides an endogenous and efficient pathway for CNS drug delivery. RMT-based approaches have shown significant potential for improving CNS drug bioavailability and enhancing the efficacy of biologics, NPs, and small molecules. However, several challenges must be addressed for clinical translation, including receptor saturation, immunogenicity, and off-target effects. Additionally, optimizing ligand specificity and transport efficiently remains critical for maximizing therapeutic outcomes while minimizing systemic exposure.

### 6.1. Transferrin Receptor (TfR)

The TfR is a transmembrane glycoprotein primarily responsible for iron uptake through the binding and endocytosis of transferrin. Two main isoforms exist: TfR1 (CD71), which is widely expressed at low levels in various tissues; and TfR2, predominantly found in hepatocytes [116,117]. The TfR is highly expressed on the luminal surface of BBB ECs, making it a key target for RMT-based CNS drug/gene delivery [118].

By conjugating therapeutic agents, such as NPs or biologics, to transferrin or TfR-targeting ligands, drug transport across the BBB can be enhanced. However, challenges such as competition with endogenous ligands, variations in receptor–ligand binding affinity, efflux transporter activity, and the endothelial glycocalyx barriers limit the efficiency of TfR-targeted delivery [116]. Additionally, the basement membrane limits the movement of drugs into the brain parenchyma. Addressing these barriers is essential to improving drug delivery to the brain, with TfR-targeted strategies emerging as a promising approach for overcoming these challenges [116].

Friden et al. showed that TfR-targeting antibodies facilitate drug transport across the BBB via RMT [119]. More recently, Lengerich et al. engineered an antibody transport vehicle (ATV) incorporating a monovalent TfR-binding site to enhance TREM2 activation in an AD mouse model, improving brain distribution, microglial function, and glucose metabolism [120]. Researchers at Denali Therapeutics developed an oligonucleotide (ASO) transport vehicle (OTV) by engineering a TfR-binding molecule for ASO delivery [121]. Studies in human TfR knockin (TfRmu/hu KI) mice and nonhuman primates (NHPs) confirmed the broad distribution of therapeutic ASOs across brain regions, highlighting the OTV’s potential for treating AD [121]. This delivery method enhanced therapeutic potential by targeting the brain broadly, including the cortex and the hippocampus. Since transferrin receptors are an entry point for ASOs, this technology has broader implications, and is not limited to CNS disorders. It also encompasses peripheral health conditions influenced by the brain, such as obesity and aging. However, the delivery system’s lack of specificity to the brain, or specific cell types within the brain, poses a limitation.

Monoclonal antibodies such as anti-transferrin receptor IgG2a (OX26), which specifically binds to TfRs, have been widely used for drug delivery across the BBB [122]. By targeting the extracellular domain of TfRs, OX26 facilitates RMT, enabling the transport of drug-loaded NPs or liposomes into the brain without interfering with the natural transferrin-binding process [123]. Studies have shown that OX26 has significantly greater uptake by brain capillary ECs than non-specific IgG2a, making it an effective vector for BBB-targeted drug delivery [124]. Pardridge et al. initially introduced this strategy in the 1990s for peptide delivery across the BBB. The approach was subsequently refined by conjugating OX26 to liposomes, enhancing drug transport efficiency into the brain [125,126,127]. Additionally, focused ultrasound (FUS) combined with microbubbles has further improved TfR-targeted liposomal delivery, increasing drug accumulation in brain ECs [128]. In Parkinson’s disease, Sela et al. designed transferrin-coated liposomes to deliver SynO4 monoclonal antibodies, effectively reducing alpha-synuclein aggregation and improving neuronal viability [129]. Similarly, Gabold et al. developed transferrin-functionalized chitosan NPs for nasal drug delivery, facilitating rapid epithelial transport and improved brain targeting, offering a promising approach for targeted drug delivery to the brain [130].

TfR-targeted nanocarriers have also shown promise in glioma therapy. Marrocco et al. engineered a ferritin-based stimuli-sensitive nanocarrier (The-0504) for glioma treatment, successfully delivering a topoisomerase 1 inhibitor (Genz-644282) and reducing tumor growth in glioma-bearing mice [131]. Sonkar et al. developed transferrin-coated gold-based theranostic liposomes co-loaded with docetaxel and glutathione-reduced gold NPs, demonstrating enhanced BBB penetration, improved drug delivery compared to Docel™, and sustained drug release, making them a promising platform for brain-targeted drug delivery and brain imaging (Table 5) [132].

The pivotal role of TfR in BBB transport has driven significant research over the past 30–35 years, significantly advancing TfR-mediated drug delivery to the CNS. Numerous preclinical studies have demonstrated the potential of TfR-targeted strategies, as highlighted in a recent comprehensive review on BBB transport of TfR-targeted NPs [133]. However, clinical translation remains challenging due to variability in receptor–ligand interactions, limited transport efficiency, and additional biological barriers within the brain microenvironment. Addressing these challenges necessitates a deeper understanding of the mechanistic aspects of TfR-mediated transport, in order to refine existing strategies and enhance the effectiveness of TfR-targeted drug delivery for neurological disorders [116].

**Table 5:** Literature review of transferrin receptor (TfR)-mediated therapies for brain delivery.

Therapeutic	Malignancy	TfR Targeting Moiety	Delivery Vehicle	Inference	Reference
Cisplatin (focused ultrasound)	Brain malignancies	OX26	Liposomes (DSPC, Cholesterol, DSPE-PEG, DSPE-PEG-maleimide, and DiD)	Significant increase in brain uptake and enhanced accumulation in hemisphere (40%)	[128]
SynO4	Parkinson’s disease	Human holo-Transferrin (T4132)	Liposomes (DPPC, cholesterol, DSPE-PEG1000-NH2, and DSPE-PEG1000-NH2)	Significant increase in brain accumulation (7-fold higher), enhanced motor function, and minimal adverse effects	[129]
Labeled β-galactosidase (for enzymatic activity)	Glioblastoma	Transferrin	Azide-functionalized chitosan NPs	Rapid target specific accumulation, and transferrin concentration was directly proportional to cellular uptake	[130]
Genz-644282	Glioblastoma	Ferritin	Nanocarrier formed with The-05 (ferritin linked with peptide that is cleavable by metalloproteases 2 and 9, and polypeptide comprising proline, alanine, serine, and glutamic acid)	Significant deposition of nanocarriers in glioma, reduced tumor burden, and enhanced survival rate	[131]
Docetaxel	Brain malignancies	Transferrin	Glutathione reduced gold NPs, incorporated in liposomes (TPGS, egg lecithin, and cholesterol)	Sustained drug release in 72 h with 2.7–4-fold higher drug concentrations in brain in comparison with marketed formulation	[132]
Lurasidone hydrochloride	Schizophrenia	Transferrin	Chitosan-modified NPs	Increased brain concentration of drug, thereby enhancing neuroprotective action	[134]
Caffeine	Strengthening physical performance	Transferrin	Liposomes (SPC, cholesterol, DSPE-PEG-MAL, and DSPE-PEG-2000)	Higher drug concentration in brain, increased dopamine release, thereby improving physical performance with targeted formulation	[135]
Curcumin	Neurodegenerative conditions	Transferrin	SLNs and nanostructured lipid carriers (cetyl palmitate, tween 60, miglyol-812, and PEG)	Controlled drug release, with 1.5-fold higher permeability with transferrin conjugation leading to higher brain uptake, and non-significant cytotoxicity. However, transferrin conjugation interfered with curcumin entrapment efficiency over time	[136]
Anti-miRNA103/107	Ischemic brain damage	Transferrin	LNPs (DSPC, cholesterol, DODAP, PEG2000-Cer16, and DSPE-PEG-Mal)	Enhanced BBB penetration of formulation over free miRNA led to significant improvement in ischemic damage repair	[137]
Docetaxel and gadolinium	Brain cancer	Transferrin	Micelles (TPGS)	Sustained drug release up to 72 h, higher brain accumulation, and enhanced therapeutic efficacy of formulation	[138]
Rivastigmine and resveratrol	Alzheimer’s disease	Transferrin	NLC (Geleol, gelucire 50/13, transcutol HP, and tween 80)	Sustained drug release, non-toxic formulation with 1.7-fold higher uptake by brain cells	[139]

1,2-distearoyl-sn-glycero-3-phosphocholine (DSPC); 2-distearoyl-sn-glycero-3-phosphoethanolamine-N-[methoxy(polyethylene glycol)-2000] (DSPE-PEG-2000); 1,1′-dioctadecyl-3,3,3′,3′-tetramethylindodicarbocyanine, 4-chlorobenzenesulfonate salt (DiD); dipalmitoyl phosphatidylcholine (DPPC); 1,2-distearoyl-sn-glycero-3-phosphoethanolamine-N-[methoxy(polyethylene glycol)] (ammonium salt) (DSPE-PEG); tocopheryl polyethylene glycol (TPGS); soy phosphatidylcholine (SPC); 1,2-distearoyl-sn-glycero-3-phosphoethanolamine-n-[poly(ethylene glycol)]-maleimide (DSPE-PEG-MAL); N-palmitoyl-sphingosine-1-succinyl[methoxy(polyethylene glycol)2000] (PEG2000-Cer16); 1,2-dioleoyl-3-dimethylammonium-propane (DODAP).

### 6.2. Insulin Receptor (IR) and Insulin-like Growth Factor-1 Receptor (IGF1R)

The insulin receptor (IR), located on the luminal surface of brain microvascular ECs, facilitates the transport of insulin and other molecules into the brain. Proteomic analyses have confirmed the presence of IR expression in brain microvessels isolated from humans, monkeys, and mice [140]. At the BBB, the IR serves two primary functions: (1) mediating the transport of circulating insulin into the brain, a mechanism with potential for targeted drug delivery; and (2) regulating BBB function, implicating insulin resistance in the pathophysiology of CNS disorders and type 2 diabetes [141]. Although the role of IR in insulin transport across the BBB is limited, IR-mediated targeting has been explored for drug delivery, offering the potential to enhance drug bioavailability while minimizing peripheral side effects by ensuring precise delivery to brain tissue. Furthermore, while the TfR remains a major target for BBB drug delivery, continued research into NP design and IR-targeting strategies is essential for developing innovative therapies for CNS disorders (Table 6). Notably, anti-receptor antibodies targeting the IR have been investigated as vectors for transporting therapeutics across the BBB.

In addition to the IR, the insulin-like growth factor-1 receptor (IGF1R) has emerged as a promising receptor for RMT delivery. This receptor facilitates the transport of IGF-1 across the BBB, and exhibits elevated expression in brain ECs compared to peripheral tissue. Alata et al. developed camelid single-domain antibodies (sdAbs, VHHs) against IGF1R, and demonstrated their ability to facilitate drug delivery via the RMT pathway [142]. Furthermore, they characterized IGF1R5, an sdAb that specifically binds to IGF1Rs at the BBB, serving as a ligand to trigger RMT, effectively delivering therapeutic cargo across the barrier [143]. Recently, insulin-fusion proteins, termed hippocampal neuron-targeting (Ht) proteins, have been engineered for protein-targeted drug delivery [144]. In vitro studies have demonstrated that insulin and Ht proteins are internalized by hippocampal neurons via insulin receptor-mediated macropinocytosis. Notably, the presence of cysteine residues is critical for efficient Ht protein delivery, with an insulin B chain mutant showing superior efficacy in transporting cargo proteins, highlighting the potential of IR- and IGF1R-mediated transport systems for CNS drug delivery (Table 6) [144].

**Table 6:** Literature examples of insulin receptor (IR) and insulin-like growth factor-1 receptor (IGF1R) used as brain targeting ligands.

Therapeutic	Malignancy	IR or IGF1R Targeting Moiety	Delivery Vehicle	Inference	Reference
Loperamide	Opioid withdrawal syndrome and nociception	Anti-insulin receptor monoclonal antibody, 29B4 (IR)	Human serum albumin NP	Therapeutic activity of drug was 3.7 times higher when conjugated with insulin and 4.4 times higher when conjugated with 29B4	[145]
Glial cell line-derived neurotrophic factor (GDNF)	Parkinson’s disease	Fusion protein (IR)	Human insulin receptor monoclonal antibody	Enabled CNS uptake of GDNF; however, was not efficacious in Parkinson’s disease model with insufficient neurons	[146]
Iduronate 2-sulfatase (enzyme)	Mucopolysaccharidosis Type II	Fusion protein (IR)	Human insulin receptor monoclonal antibody	Enabled CNS uptake of iduronate 2-sulfatase	[147]
BMSC and insulin-like growth factor-1	Cerebral ischemia	Insulin-like growth factor-1 (IGF1R)	BMSC treated with insulin-like growth factor-1	Improved survival migratory function of BMSC, with significant improvement in cerebral blood flow and behavioral outcomes	[148]
IGF1R3, IGF1R4, and IGF1R5	Brain malignancies	Anti-IGF1R antibodies (IGF1R3, IGF1R4, and IGF1R5; IGF1R)	Free single-domain anti-IGF1R antibodies	Significant, saturable accumulation of non-permeable antibodies in brain via IGF1 receptor	[142]
Caffeic acid	Diabetic neuropathy	Caffeic acid (IGF1R)	Free drug	Reduced inflammation and oxidative stress and acted as potential target for IGF1R	[149]
LR3-IGF-1	Alzheimer’s disease	LR3-IGF-1 (IGF1R)	Free peptide	Enhanced actin remodeling, improved restored body composition, and reduced filamentous plaques; however, no improvement in cognitive functions	[150]
Acrylic acid	Neural disease (IGF1R)	Insulin-like growth factor-1	Polyethylene-glycol diacrylate microparticles conjugated with acrylic acid	Stable formulation with sustained release of IGF1R, acts as dual delivery system, and has potential to restore health of injured neurons	[151]

Bone marrow mesenchymal stem cell (BMSC); insulin-like growth factor-1 (IGF1); ling arginine 3-insulin-like growth factor-1 (LR3-IGF-1).

### 6.3. Lipoprotein Receptor-Related Proteins

The low-density lipoprotein receptor (LDLR) family of cell membrane glycoproteins plays a crucial role in lipid homeostasis, energy metabolism, and synthesis of the cell membrane and hormones. This family comprises LDLRs, very-low-density lipoprotein receptors (VLDLRs), and low-density lipoprotein-related protein receptors (LRP), including, LRP1, LRP2 (megalin), LRP5, LRP6, and LRP8 (apoER2) [152]. The LDLR primarily interacts with a cholesteryl ester containing a lipoprotein particle, low-density lipoprotein (LDL), forming an LDL-LDLR complex at neutral pH. LDLRs are detected on the ECs of brain capillaries; however, other members of the LDLR family, including the LDLR, LRP1, and LRP2, are also expressed on astrocytes facilitating RMT [153,154]. In the systemic circulation, other circulating lipoprotein particles, including apolipoprotein E (apoE) and very-low-, intermediate-, and high-density lipoproteins, also exhibit affinity towards LDLRs [155]. The substrate–receptor complex is internalized by endosomes via the clathrin-dependent pathway, followed by lysosomal hydrolysis, where the lipid is dissociated from the complex due to a low pH, and the receptor is recycled back to the cell surface [156,157]. The transcription of LDLRs in the nucleus regulates cholesterol [157]. The function of LDLRs is well understood in cardiovascular diseases, cholesterol homeostasis, and atherosclerosis. However, despite the critical role of lipids in myelin synthesis, the metabolism of lipoproteins in the central nervous system remains vague [158]. While most of the lipids required for the normal functioning of the brain are endogenously synthesized via de novo synthesis, some brain uptake of lipoproteins, fatty acids, and cholesterol from the systemic circulation suggests the presence of lipid transporters. Dysregulated lipoprotein metabolism in brain endothelia is linked with various chronic neurological disorders. For example, the endocytosis of apolipoprotein E (apoE), which binds to LDLRs, influences the accumulation and the clearance of amyloid *β* peptide, a key factor in the progression of Alzheimer’s disease [153,159]. Additionally, LRP5 and LRP6 are involved in the Wnt/*β* catenin signaling pathway, elucidating its importance in gliomas and medulloblastomas [160].

LRP1 and LRP2, integral members of the LDL receptor family, are highly expressed in the BBB, making them attractive targets for drug delivery systems designed to facilitate brain penetration. Given their elevated expression in glioma cells, LRP ligands are also employed for brain tumor therapy. LRP1 mediates ligand internalization, contributes to BBB disruption following ischemic events, regulates tight-junction proteins, and facilitates the clearance of extracellular matrix (ECM)-degrading proteinases [161]. Leveraging LRP1 and LRP2 for drug delivery involves the conjugation of therapeutic agents or NPs with ligands, peptides (such as Angiopep-2), or antibodies that specifically bind to these receptors, thereby promoting transcytosis into the brain parenchyma. This strategy is particularly beneficial for treating neurodegenerative diseases, brain tumors, and lysosomal storage disorders, as it enhances drug delivery efficiency while minimizing peripheral distribution and potential toxicity [162]. For example, Guo et al. developed statin-loaded Angiopep-2-anchored NPs (S@A-NPs), which selectively upregulate LRP1 expression in both brain microvascular ECs and brain metastatic tumor cells [163]. These NPs efficiently and self-promotingly cross the BBB and target brain metastases via Angiopep-2-mediated endocytosis, presenting a promising approach for the clinical management of brain metastasis [163]. Additionally, functionalized LNPs incorporating peptides that target receptors overexpressed on brain ECs and neurons—such as RVG29, T7, AP2, and mApoE—have been demonstrated to enhance mRNA transfection in the mouse brain, while reducing hepatic accumulation following systemic administration [164].

Functionalizing lipidic, polymeric, and metallic NPs with LDLR substrates—such as apoE, apolipoprotein B (apoB), Angiopep-2, polysorbate-80, and lecithin—further enhances brain uptake via RMT [165]. Studies have shown that functionalization of liposomes with apoE or its mimetics enhances the likelihood of NPs undergoing endocytosis by LDL receptors on the brain endothelium [165,166]. Seo et al. synthesized apolipoproteinB29-conjugated (apoB29) gold NPs (ApoB@AuNP), which exhibited a 20-fold increase in astrocyte uptake compared to non-targeted NPs, enhancing reactive oxygen species production and thereby improving therapeutic efficacy [167]. Another emerging approach involves modifying exosomes for targeted brain delivery. While unmodified exosomes tend to accumulate in the spleen and liver, apoE conjugation redirects their uptake to the brain and glioblastoma cells, improving treatment outcomes [168]. These studies lay the groundwork for the development of new treatment strategies for brain disorders.

Clinical trials (NCT01967810, NCT01480583, NCT02048059, and NCT01497665), such as those investigating ANG1005 and GRN1005, have explored LRP1-targeting therapeutics, where paclitaxel molecules are covalently linked to Angiopep-2 to enhance BBB penetration and tumor uptake while circumventing P-gp-mediated drug efflux (Table 7) [169,170]. Given that LDLRs are overexpressed in rapidly proliferating tumor cells, they represent promising targets for brain cancer therapy. Many LDLR substrates are biocompatible and biodegradable, facilitating efficient drug transport across the BBB. However, as LDLRs are known to be expressed in normal tissues, off-target effects and systemic toxicity remain concerns, potentially reducing the drug concentration at the intended site. Despite these challenges, LDLR-targeted strategies hold significant promise for treating neurological disorders and brain malignancies [165].

### 6.4. Neonatal Fc Receptor (FcRn)

The FcRn, also known as the Brambell receptor, is responsible for binding to the Fc region of immunoglobulin G (IgG) and to albumin, two of the most abundant proteins in the systemic circulation. It is predominantly expressed in the brain microvascular endothelium and the choroid plexus epithelium, where it plays a crucial role in extending the half-life of IgG and albumin by recycling them, rather than degrading them. The FcRn mediates both efflux (from the brain to the bloodstream) and potentially influx (from the bloodstream to the brain) transcytosis of IgG, which may have significant implications for the delivery of drugs to the CNS [178]. Studies have demonstrated that the FcRn facilitates the rapid efflux of IgG from the brain parenchyma back into the bloodstream, a process known as “reverse transcytosis”, which may limit the therapeutic efficacy of antibodies targeting the brain [179]. Consequently, understanding the FcRn’s role in IgG transport across the BBB has led to efforts to modulate antibody–FcRn interactions to either enhance or reduce brain delivery of therapeutic antibodies [178]. The FcRn binds to the Fc portion of IgG in a pH-dependent manner, preventing IgG from degradation and facilitating its transcellular transport. This interaction is characterized by high affinity at acidic pH (~6.0–6.5), which allows the FcRn to bind IgG. However, as the environment becomes more neutral (~7.4), the receptor’s affinity decreases significantly, leading to the release of IgG back into the circulation [180]. In addition to IgG, the FcRn also binds to albumin under mildly acidic conditions, which plays a crucial role in extending albumin’s half-life in the bloodstream to approximately 21–28 days [181]. This interaction helps to maintain albumin’s high plasma concentration (35–55 g/L) by recycling it through endosomal pathways and protecting it from degradation [182].

FcRn-targeted drug delivery platforms involve the engineering of therapeutic antibodies or fusion constructs with optimized Fc regions that enhance their interaction with FcRns and promote transcytosis into the brain. Engineered antibodies with modified Fc domains showed improved transport across the BBB in a mouse model [178]. Recent studies have further explored this pathway to enhance the brain delivery of various therapeutics. Additionally, Haqqani et al. reviewed RMT mechanisms for brain delivery of therapeutics, emphasizing the significance of receptors like the FcRn in facilitating this transport [115]. Holst et al. investigated the subcellular trafficking and transcytosis efficacy of various receptor types, including the FcRn, for therapeutic antibody delivery at the BBB, providing insights into optimizing such delivery systems [182]. Simonneau et al. utilized BBB organoid arrays to study receptor-mediated antibody transcytosis, highlighting the FcRn’s potential for mediating drug delivery across the BBB [183]. Furthermore, Mhaske explored receptor-assisted nanotherapeutics for overcoming the BBB, underscoring the role of the FcRn in enhancing drug delivery to the brain [184]. Albumin, another FcRn ligand, has been extensively explored as a drug delivery carrier, due to its favorable properties. Albumin-based NPs are particularly attractive, due to their excellent biocompatibility, low immunogenicity, and prolonged systemic circulation. Their extended half-life is primarily attributed to FcRn-mediated recycling, which protects albumin from lysosomal degradation via a pH-dependent retrieval mechanism [185]. In glioma models, albumin NPs functionalized with the cell-penetrating peptide have demonstrated enhanced brain penetration and tumor-targeting capabilities [186]. Additionally, the free thiol group of cysteine residue (Cys34) on albumin’s domain I has been employed in drug conjugation strategies, such as the Drug Affinity Complex (DAC) approach. A notable example is Aldoxorubicin, an acid-sensitive doxorubicin prodrug that binds covalently to albumin via Cys34 following intravenous administration [187]. This conjugate has shown promise in clinical trials for the treatment of sarcoma and glioblastoma [187] (Table 8).

These findings highlight FcRn’s emerging role in CNS drug delivery, and the potential for optimizing Fc engineering and NP formulations to enhance transcytosis efficiency. Additionally, a comprehensive review of FcRn-IgG immunobiology, including its functional and pathological roles, along with an overview of FcRn-targeted therapy development, provides valuable insights into the advancing field [188].

**Table 8:** Strategies to target the neonatal Fc receptor (FcRn) for brain-targeted drug delivery.

Therapeutic	Malignancy	FcRn Targeting Moiety	Delivery Vehicle	Inference	Reference
IgG1 N434A and IgG1 H435A	Brain malignancies	IgG1 N434A (high-affinity) and IgG1 H435A (low-affinity)	Free monoclonal antibodies	N434A and H435A have affinity towards efflux transporter FcRn, making them potential targeting moieties for brain delivery	[189,190]
Anti-PD-L1 IgG	Brain glioblastoma	FcRn high-affinity ^89^Zr-DFO-C4 with FSU	C4 radioligands	Leveraging FcRn confers improved kinetic properties to ^89^Zr-DFO-C4	[191]
Doxorubicin	Glioma	Albumin	Acid-sensitive prodrug	Showed promise in clinical trials for treatment of sarcoma and glioblastoma	[187]

Immunoglobulin-G (IgG1), focused ultrasound (FSU).

## 7. Conclusions

TMT and RMT represent a promising and potential transformative approach to overcoming the challenges of drug delivery across the BBB. Significant progress has been made in understanding the biology of RMT/TMT and its applications in treating CNS disorders. However, challenges such as receptor or transporter saturation, immunogenicity, and cargo limitations must be addressed to achieve widespread clinical adoption. Continued advancements in ligand design, nanotechnology, and personalized medicine will be instrumental in realizing the full therapeutic potential of utilizing RMT/TMT and improving outcomes for patients with neurological diseases. With ongoing advancements in drug design, NP engineering, and transporter and receptor biology, TMT/RMT are poised to play a central role in the development of next-generation therapies for the treatment of CNS disorders. Continued research and innovation will be crucial to unlocking their full potential and bringing TMT/RMT-based therapies closer to clinical reality.

## Figures and Tables

**Table 1:** Literature review of glucose transporter-1 (GLUT-1)-targeted therapies for brain delivery.

Therapeutic	Malignancy	GLUT-1 Targeting Moiety	Delivery Vehicle	Inference	Reference
Brain-derived neurotrophic factor gene	Alzheimer’s disease	Mannose	Liposomes	Increase in synaptophysin in patients with Alzheimer’s disease	[60]
Theranostic application	Glioblastoma	Glucuronic acid	Iron oxide NPs	Efficacious delivery system with significant delay in tumor growth	[69]
Cisplatin	Glioblastoma	Chitosan oligosaccharide	Magnetite NPs with arginine modification	Significantly induced tumor cell apoptosis and reduced tumor burden	[70]
Boron-dipyrromethane (fluorescent dye)	Epilepsy	2-deoxy-2-amino-D-glucose modified DSPE-PEG2000	Modified DSPE-PEG2000 polymeric NPs	Enhanced cellular uptake by capillary endothelial cells and thereby increased brain accumulation	[71]
Cannabidiol and brain-derived neurotrophic factor	Alzheimer’s disease	Mannose-modified chitosan	Chitosan-coated poly d,l-lactic-co-glycolic acid polymeric NPs	Prolonged drug release, biocompatible and non-toxic NPs, and 4-fold increase in therapeutic concentration in targeted vs. non-targeted NPs	[72]
Lacosamide	Epilepsy	Glucose	Gold NPs	Attained therapeutic concentration, reduced seizure activity, and dose reduction	[73]
Docetaxel	Glioblastoma	Glucose-conjugated cholesterols	Liposomes	Improved brain accumulation of docetaxel	[64]
3D6 antibody fragments	Alzheimer’s disease	Glucose	Micelles	41-fold-enhanced 3D6-Fab accumulation in the brain	[65]

**Table 3:** Literature review of monocarboxylate Transporter 1 (MCT1)-targeted therapies for brain delivery.

Therapeutic	Malignancy	MCT1 Targeting Moiety	Delivery Vehicle	Inference	Reference
Carmustine and temozolomide	Glioblastoma	β-hydroxybutyric acid	SLN	Higher drug uptake in targeted SLNs and increased tumor cell apoptosis	[99]
AZD3965	Solid tumors	AZD3965 (MCT1 inhibitor)	PEG-b-(poly(dipropylaminoethyl methacrylate), ultra-pH-sensitive micelles	Released drug at acidic pH, dose reduction (>200-fold), reduced toxicity, and increased T-cell infiltration	[101]
AZD3965	Advanced cancer	AZD3965 (MCT1 inhibitor)	Free drug	On-target activity with grade I/II adverse effect and reversible ocular changes	[103]

**Table 4:** Literature review of organic anion-transporting polypeptide (OATP)-targeted therapies for brain delivery.

Therapeutic	Malignancy	OATP Targeting Moiety	Delivery Vehicle	Inference	Reference
Ketoprofen, flurbiprofen, naproxen, and salicylic acid	Glial cells	3,5-diiodo-l-tyrosine and T4 (OATP1C1)	Prodrug	Showed binding with OATP1A4/1A5/1A5, along with OATP1C1	[112]
Paclitaxel	Peripheral neuropathy	Glycyrrhizic acid (OATP1A1 and OATP1B2 inhibitor)	Co-administration	Improved behavioral and electrophysiological outcomes	[113]
Edaravone	Cerebral ischemia–reperfusion	Dexborneol (OAT1 and OAT3)	Co-administration	Significant reduction in injury, upregulated expression of influx transporters (OAT1 and OAT3)	[114]

**Table 7:** Strategies to target low-density lipoprotein receptors (LDLR) for brain-targeted drug delivery.

Therapeutic	Malignancy	LDLR Targeting Moiety	Delivery Vehicle	Inference	Reference
Pristine proton beam	Glioblastoma malforms	ApoB (LDLR)	ApoB-conjugated gold NPs	Preferential invasion of NPs in tumor microenvironment and significant reduction in tumor volume	[167]
DHODH inhibitor and siRNA (siGPX4)	Glioblastoma	ANG2 (LRP-1)	ANG2 peptide-modified drug-loaded exosomes in combination with magnetite particles	Synergism was observed, surface-modified exosomes recognized LRP1 receptors on BBB and tumor cells, achieved localized accumulation to create targeted magnetic field, and displayed biocompatibility	[168]
Fluorescent-dye load (as model drug)	CNS malignancies	Lecithin (LDLR)	Cholesteryl oleate NPs	Lecithin enabled adsorption of apoE and mediated BBB penetration	[171]
Laser exposure (phototherapy)	Glioblastoma multiforme	ANG2 (LRP-1)	Gold nanorods	Higher uptake of nanorods led to increased production of ROS under laser irradiation, higher apoptosis and autophagy	[172]
DHA and indocyanine green	Glioblastoma	Lactoferrin (LRP-1)	Self-assembling NPs comprising DHA and indocyanine green	Proposed non-chemotherapeutic treatment option, suppressed progression of tumor with minimal adverse effects, and improved survival in mouse model of glioblastoma	[173]
Paclitaxel	High-grade glioma	ANG2 (LRP-1)	Covalent linkage of paclitaxel to ANG2 (ANG1005)	Increased brain uptake of paclitaxel, achieving therapeutic brain concentrations in phase 1 clinical trial; no significant improvement in efficacy was observed by end of phase II clinical trial (NCT01967810)	[169,174]
BB25 (*γ*-secretase modulator)	Alzheimer’s disease	mLRP1_DIV (LRP1 antibody)	Liposomes loaded with BB25	Successful use of mLRP1_DIV as artificial LRP1 substrate, enhanced uptake of liposomes, and reduced toxic amyloid *β*42 peptide levels	[175]
Docetaxel	Glioblastoma	ANG2 (LRP-1)	Zein protein conjugated with PEG and ANG2, forming polymeric NPs	Four-fold increase in BBB penetration and conserved cytotoxicity of drug, suggesting release of drug from NPs	[176]
Paclitaxel	Glioblastoma	A*β*-CN (LRP-1)	Nanomicelle-decorated A*β*-CN (modified amyloid protein to reduce cytotoxicity)	A*β*-CN mediated adsorption of ApoE, acted as substrate of LDLR and LRP1, and facilitated brain-targeted delivery	[177]

Low-density lipoprotein receptor (LDLR); low-density lipoprotein-related protein receptor (LRP); apolipoproteinB29 (ApoB); ANGIOPEP-2 (ANG2); reactive oxygen species (ROS); polyethylene glycol (PEG); amyloid *β* peptide-CN (A*β*-CN); dihydroartemisinin (DHA).
